# Exploratory analysis of the effect of a controlled lifestyle intervention on inflammatory markers – the Healthy Lifestyle Community Programme (cohort 2)

**DOI:** 10.1186/s40795-023-00684-2

**Published:** 2023-02-06

**Authors:** Christian Koeder, Corinna Anand, Sarah Husain, Ragna-Marie Kranz, Nora Schoch, Dima Alzughayyar, Norman Bitterlich, Andreas Hahn, Heike Englert

**Affiliations:** 1 Institute of Food Science and Human Nutrition, Leibniz University Hanover, Hanover, Germany; 2 Department of Nutrition, University of Applied Sciences Münster, Münster, Germany; 3 Department of Biostatistics, Medizin & Service GmbH, Chemnitz, Germany

**Keywords:** Plant-based diet, Subclinical inflammation, C-reactive protein, Homocysteine, Adiponectin, Cardiovascular disease, Lifestyle intervention

## Abstract

**Background:**

Chronic low-grade inflammation is associated with an increased risk of chronic disease and mortality. The objective of the study was to test the effect of a healthy lifestyle intervention on biomarkers of inflammation (among other risk markers).

**Methods:**

We conducted a non-randomized controlled trial with mostly middle-aged and elderly participants from the general population in rural northwest Germany (intervention: *n* = 114; control: *n* = 87). The intervention consisted of a 1-year lifestyle programme focusing on diet (largely plant-based; strongest emphasis), physical activity, stress management, and social support. High-sensitivity C-reactive protein (hs-CRP) was assessed at baseline, 10 weeks, 6 months, and 1 year. Homocysteine (Hcy) was assessed at baseline, 10 weeks, and 1 year. Adiponectin (Apn) was assessed at baseline and 10 weeks. An exploratory analysis of these inflammatory markers assessing the between-group differences with ANCOVA was conducted.

**Results:**

The 1-year trajectory of hs-CRP was significantly lower in the intervention group compared to control (between-group difference: -0.8 (95% CI -1.2, -0.3) mg/l; *p* = 0.001; adjusted for baseline). The 1-year trajectory of Hcy was non-significantly higher in the intervention compared to control (between-group difference: 0.2 (95% CI -0.3, 0.7) µmol/l; *p* = 0.439; adjusted for baseline). From baseline to 10 weeks, Apn decreased significantly more in the intervention group compared to control (between-group difference: -1.6 (95% CI -2.7, -0.5) µg/ml; *p* = 0.004; adjusted for baseline).

**Conclusions:**

Our study shows that healthy lifestyle changes can lower hs-CRP and Apn levels and are unlikely to significantly affect Hcy levels within 1 year.

**Trial registration:**

German Clinical Trials Register (DRKS; reference: DRKS00018775, registered 12 Sept 2019; retrospectively registered; www.drks.de).

**Supplementary Information:**

The online version contains supplementary material available at 10.1186/s40795-023-00684-2.

## Background

Chronic low-grade inflammation is associated with an increased risk of a variety of chronic diseases, including cardiovascular disease (CVD) and cancer [1]. High-sensitivity C-reactive protein (hs-CRP) is an established peripheral biomarker of inflammation [2]. For every 1 mg/l increase in hs-CRP there appears to be an increase in all-cause mortality by > 30% in men and > 15% in women [1]. Higher hs-CRP levels are associated with increased inflammation and oxidative stress which are in turn associated with impaired endothelial function and increased CVD risk [3].

Shifting dietary patterns towards a healthy, largely plant-based diet [4] would likely decrease hs-CRP levels [5]. Most prominently, a traditional Mediterranean diet is associated with lower inflammatory markers, including lower hs-CRP levels [3, 6]. Similarly, vegetarian dietary patterns are associated with significantly lower hs-CRP values (~ 0.6 mg/l lower compared to non-vegetarian diets) [7]. In addition, increasing physical activity levels has been shown to lower hs-CRP [2]. While hs-CRP is the most commonly assessed inflammatory marker, other inflammatory markers may also be of particular interest in connection with plant-based dietary interventions.

Homocysteine (Hcy) serves as a functional marker of vitamin B12, folate/folic acid, and vitamin B6 status, and a deficiency in any of these vitamins is associated with increased Hcy. In individuals with obesity [8] or hypothyroidism [9], Hcy levels are typically increased. Frequently, Hcy levels positively correlate with hs-CRP levels, and Hcy has been shown to stimulate CRP expression via downregulation of peroxisome proliferator-activated receptor γ (PPARγ) [10], a transcription factor with an important role in regulating glucose and lipid metabolism [11]. Increases in hs-CRP [12] and Hcy [13] are both associated with impaired insulin sensitivity, and increased Hcy levels are associated with an increased risk of hypertension and arterial wall damage [14]. Furthermore, Hcy is linearly associated with stroke risk, with each 1 µmol/l increase in Hcy being associated with a 6% increase in stroke risk [15]. When the recommendation of a predominantly plant-based diet is given, vitamin B12 intake is likely to decrease (unless fortified foods or supplements are consumed), with a probable increase in folate intake and an adequate intake of vitamin B6 [16]. In this context, due to a decrease in vitamin B12 intake, an increase in Hcy may occur [17].

Adiponectin (Apn) is a hormone secreted by adipose tissue and a controversial inflammatory marker [18]. Higher Apn levels are frequently interpreted to be beneficial, although conflicting results have been reported [18]. Higher Apn levels have been shown to be associated with increased insulin sensitivity, decreased oxidative stress, decreased inflammation, inhibited release of tumor necrosis factor α (TNF-α) and interleukin-6 (IL-6) as well as decreased activation of nuclear factor κB (NF-κB) [19]. In contrast, higher Apn levels are associated with increased all-cause mortality in haemodialysis patients [20] and individuals with heart disease [21]. Similarly, in individuals with a history of ischaemic stroke, higher Apn levels are associated with an increased risk of having another ischaemic stroke [22]. In addition, Apn levels are increased in a variety of chronic inflammatory diseases, including rheumatoid arthritis, chronic kidney disease, type 1 diabetes, and irritable bowel syndrome [23].

Inverse associations of Apn and hs-CRP have been documented [24–27], while some (but not all) studies also indicate an inverse association between Apn and Hcy [28–30]. Like in the case of Hcy, the potential effect of plant-based diets on Apn levels are unclear [31]. To date, controlled trials assessing the effect of healthy lifestyle changes including a predominantly plant-based diet on Apn levels in clinically healthy participants from the general population are lacking [31, 32].

Against this background, we hypothesized that our lifestyle intervention would lead to improvements in inflammatory markers (among other risk markers). The objective of the study was to test the effectiveness of the intervention in this regard.

## Methods

### Study design

We conducted a non-randomized, controlled intervention trial, with measurement time points at baseline, 10 weeks, 6 months, and 1 year. Hcy was not assessed at 6 months (as we did not expect such a short-term effect on vitamin B12 status). Apn was only assessed at baseline and 10 weeks (as a potential effect on Apn was uncertain, as not all time points could be assessed for financial reasons, and as the strongest effect was expected after the intensive phase of the programme, i.e. after 10 weeks). The study was intended to last 2 years, but due to the COVID-19 pandemic the last two time points could not be included in the present analyses: the 1½-year time point was not included because, due to the pandemic, there was a time delay of the assessment of the control group (20 instead of 18 months). The 1½-year time point, however, was included in sensitivity analyses. Due to the pandemic, there were no assessments at the 2-year time point in the control group (the 1½-year and 2-year time points only concern hs-CRP as Hcy and Apn were not assessed at these time points).

The intervention consisted of a healthy lifestyle programme, and the control group received no intervention (as described previously [33]). We followed guidelines for non-randomized controlled trials [34]. The control group study arm started 6 months later (October 2018) than the intervention group (April 2018), with equivalent follow-up durations, because funding was granted at short notice and for a specific time period and there were insufficient capacities to recruit and start both study arms at the same time.

### Participants

Participants were recruited from the general population (see the inclusion criteria below) at local public events in two separate small municipalities in northwest Germany (recruitment for the intervention group: at an information event about the intervention, incorporated into the regular weekly market, Billerbeck, North Rhine-Westphalia, February 2018; recruitment for the control group: at the annual horticultural show, Legden, North Rhine-Westphalia, September 2018 [33]). In each town, the mayor and a local physician helped with the recruitment of local citizens at these events. Furthermore, leaflets, posters, and an announcement in the local newspaper were used to recruit additional participants in each municipality. The only inclusion criteria were the physical and mental ability to take part in the study and to be ≥ 18 years old. A total of 114 and 87 participants were recruited for the intervention and control groups, respectively.

### Lifestyle programme

The lifestyle intervention (Healthy Lifestyle Community Programme, cohort 2) consisted of an intensive phase (first 10 weeks) and a less intensive phase (from 10 weeks until the end of the study). The intensive phase consisted of 14 seminars and 8 workshops [33, 35, 36]. The less intensive phase consisted of monthly seminars. Participants of the intervention group also took part in two one-on-one lifestyle coaching sessions (at baseline and 10 weeks) and received a healthy lifestyle handbook, a recipe booklet, and a laminated information sheet with an overview of the lifestyle recommendations [36].

The intervention programme and materials addressed healthy lifestyle choices in terms of diet, physical activity, stress management, and community support. The strongest emphasis was on dietary change. Dietary recommendations were to move towards a healthy, predominantly plant-based diet, i.e. to increase the intake of fruit, vegetables, whole grains, legumes (including soya foods), nuts, seeds, and healthy oils and to decrease the intake of meat, eggs, butter, full-fat dairy, added sugars, refined grains, and salt as well as to avoid alcohol excess. Recommendations regarding physical activity, stress management, and community support were not specific but included suggestions to walk and cycle more, form a walking or jogging group with other participants, identify an enjoyable way to exercise regularly, establish short daily “relaxation rituals”, practice mindfulness, spend more time in nature, and to form additional support groups with other participants (for example, for cooking and eating together).

### Assessment of parameters

Biomarkers were assessed from blood samples. All blood samples were taken in the morning (6:00 to 11:00 am) and in the fasted state. Laboratory assays are shown in Supplementary table 1, Additional file 1. Dietary intake was assessed with semi-quantitative 3-day food protocols. Adherence to dietary recommendations was assessed using the plant-based diet index (PDI), healthful PDI (hPDI), and unhealthful PDI (uPDI) by Satija et al. [4]. Physical activity (in categories) and socio-demographic data and were assessed with questionnaires.

### Study hypotheses

In terms of hs-CRP, Hcy, and Apn, the study hypotheses were that the intervention would significantly decrease hs-CRP (within-group and compared to control; from baseline to 10 weeks and from baseline to 1 year), that the intervention would increase Apn (within-group and compared to control; from baseline to 10 weeks) and that the intervention would not increase Hcy (within-group and compared to control; from baseline to 10 weeks and from baseline to 1 year). The three main hypotheses were regarding the between-group changes (hs-CRP and Hcy: 1-year changes; Apn: 10-week changes). Any detected differences in the secondary end points hs-CRP, Hcy, and Apn are considered exploratory.

### Statistical analyses

A sample size calculation was performed based on changes in body weight (the primary outcome measure of the study [33, 35]) on which the sample size was based. However, for the secondary end point of hs-CRP change (from baseline to 1 year) an additional power calculation was performed (using data from a comparable study [37]), to estimate statistical power with the given sample size. Based on the expectation of a hs-CRP decrease of ~ 30% from baseline to 1 year in the intervention group (effect size: ∼0.38) [37], and no change in the control group, our sample size was adequate to detect a difference in hs-CRP change with a power of 0.65 and at a significance level of 0.05. Holm-Bonferroni correction was conducted to adjust for multiple comparisons.

Fisher’s exact test was used for between-group comparisons of categorical variables. Independent t-test was used for normally distributed and Mann–Whitney U test for non-normally distributed continuous variables. Shapiro–Wilk test was used to assess data for non-normality (*p* < 0.05 was defined as describing a non-normal distribution). To evaluate within-group changes, paired t-test and Wilcoxon signed-rank test were used for normally and non-normally distributed variables, respectively. All tests were two-sided.

For the analyses of changes from baseline to 10 weeks, between-group differences were assessed with a one-way analysis of covariance (ANCOVA). For between-group comparisons of 1-year trajectories, a repeated measures ANCOVA was used, with potential confounders as covariates.

Bivariate correlations were assessed with Spearman’s rho correlations (two-sided). Analyses were based on unimputed data (complete case analysis, CCA). In sensitivity analyses imputed data (last observation carried forward, LOCF) were used. All analyses were conducted using IBM SPSS Statistics (Version 25.0. Armonk, NY). Participants with an infection or common cold (self-reported at either measurement time point) were excluded from hs-CRP analyses but were then included again in sensitivity analyses.

## Results

### Baseline characteristics

**Fig. 1:**
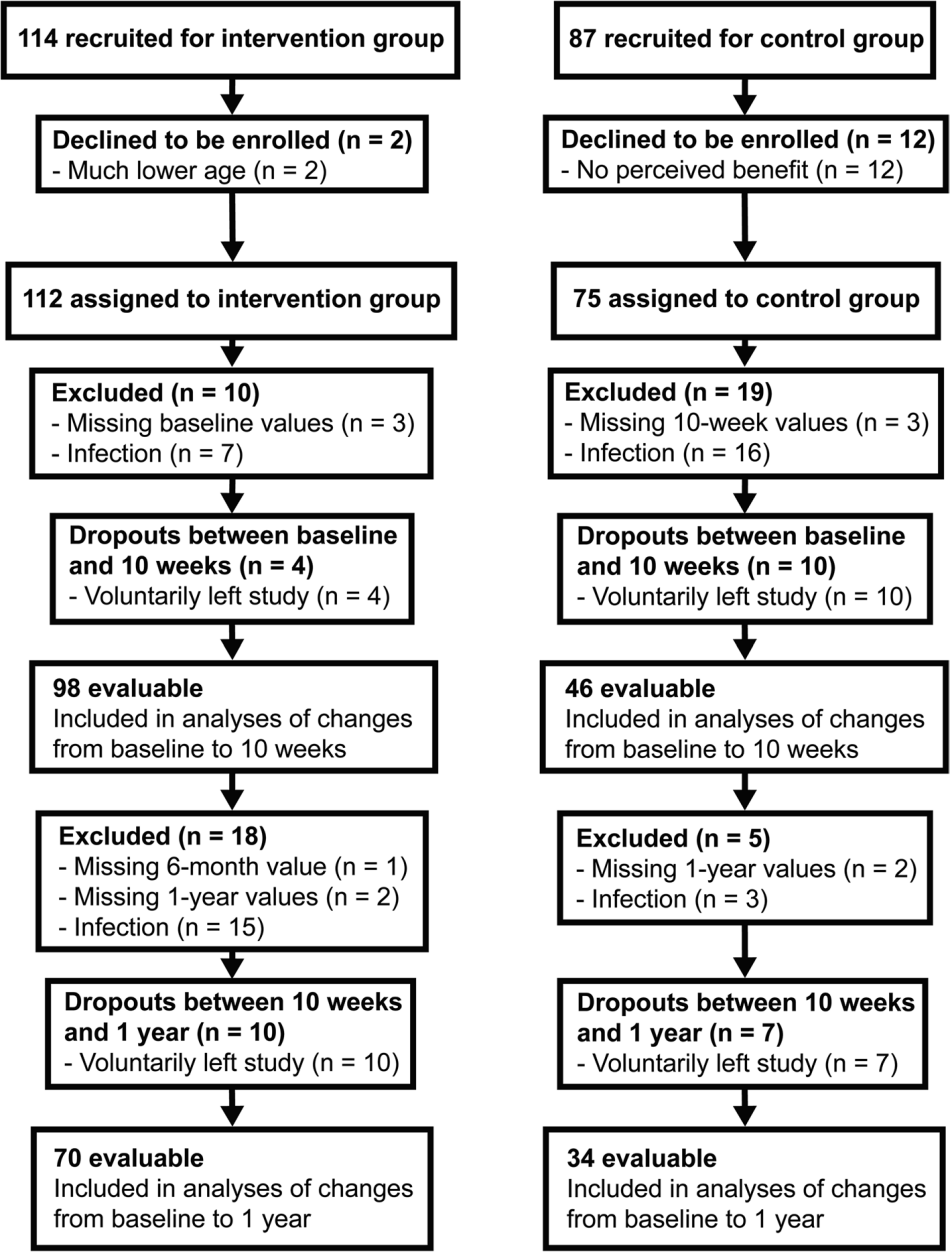
Flow chart of participants through the study (hs-CRP analysis) hs-CRP: high sensitivity C-reactive protein

For the analysis of hs-CRP changes (1-year trajectories), a total of 104 participants (intervention: 70; control: 34) were available (Fig. 1). The analysis of Hcy changes (1-year trajectories) is based on a total of 120 participants (intervention: 68; control: 52). For the analysis of Apn changes (baseline to 10 weeks), a total of 141 participants (intervention: 80; control: 61) were available (Supplementary Fig. 1, Additional file 1).

At baseline and compared to control, the intervention group had a higher age (*p* = 0.003), higher Apn levels (*p* = 0.001), and a higher (more favourable) hPDI (*p* = 0.006). Categories of educational levels were significantly different between groups (with neither group having a clearly higher education; *p* = 0.009; Table 1). There were no significant differences in PDI (*p* = 0.553), uPDI (*p* = 0.069), or other baseline characteristics (Table 1), including alcohol intake frequency and the percentage of participants with any of a variety of diagnosed disease conditions (as described previously[33]). Furthermore, there were no significant between-group differences in terms of the percentage of participants with a history of stroke, a history of cancer, a family history (siblings, parents, grandparents) of myocardial infarction or stroke, or the percentage of participants who (based on baseline values) had hypertension, high total cholesterol (TC), LDL-C, non-HDL-C, triglycerides (TAG), HbA1c, or low HDL-C.

**Table 1:** Baseline characteristics of evaluable participants (CCA)

**Characteristics**		**Intervention** (*n* = 70)		**Control** (*n* = 34)		***p*-value**
		**Means** **or n**	**SEM** **or %**	**Means** **or n**	**SEM** **or %**	
Men, n (%)		24	34.3	13	38.2	0.827 ^a^
Age at baseline, years		59.7	1.0	53.9	1.7	**0.003** ^b^
hs-CRP, mg/l		1.3	0.2	2.6	0.7	0.667 ^b^
Hcy, µmol/l *		12.4	0.4	11.8	0.4	0.635 ^b^
Apn, µg/ml †		10.5	0.6	7.7	0.6	**0.001** ^b^
Body weight, kg		81.4	2.0	82.3	3.7	0.936 ^b^
BMI, kg/m^2^		27.2	0.5	27.5	1.1	0.771 ^b^
WC, cm		98.7	1.6	94.5	2.6	0.162 ^c^
Overweight, n (%)		49	70.0	21	61.8	0.504 ^a^
Obese, n (%)		16	22.9	8	23.5	1.000 ^a^
Smoker status, n (%)	Never	41	58.6	17	50.0	0.262 ^a^
	Ex	23	32.9	10	29.4	
	Smoker	6	8.6	7	20.6	
Marital status, n (%)	Married	59	84.3	31	91.2	0.945 ^a^
	Partner (unmarried)	5	7.1	1	2.9	
	Single (not widowed)	3	4.3	1	2.9	
	Single (widowed)	3	4.3	1	2.9	
Educational level, n (%)	Lower secondary school	15	21.4	11	32.4	**0.009** ^a^
	Secondary school	32	45.7	10	29.4	
	University entrance qualification	10	14.3	12	35.3	
	University degree	13	18.6	1	2.9	

*CCA* Complete case analysis, *hs-CRP* high-sensitivity C-reactive protein. *Hcy* Homocysteine, *Apn* Adiponectin, *BMI* body mass index, *WC* Waist circumference, *SEM* Standard error of the mean, *p*-value for between-group comparisons by:

^a^ Fisher’s exact test (two-sided)

^b^ Mann–Whitney U test (two-sided)

^c^ independent t-test (two-sided)

* Intervention: n = 68; control: n = 52; † Intervention: n = 80; control: n = 61

### Changes in hs-CRP (baseline to 10 weeks)

From baseline to 10 weeks, hs-CRP significantly decreased in the intervention group (-0.5 [95% CI -0.9, -0.1] mg/l; *p* < 0.001; *n* = 98), with no significant changes in the control group (0.3 [95% CI -0.6, 1.2] mg/l; *p* = 0.956; *n* = 46). This constituted a between-group difference in hs-CRP changes of -1.0 (95% CI -1.7, -0.3) mg/l (*p* = 0.006; adjusted for baseline). Results were confirmed in sensitivity analyses (Additional file 1, Supplementary table 2).

### Changes in hs-CRP (baseline to 6 months)

From baseline to 6 months, hs-CRP significantly decreased in the intervention group (*p* = 0.002; *n* = 83) and non-significantly increased in the control group (*p* = 0.905; *n* = 42). The 6-month trajectory of hs-CRP was significantly lower in the intervention group compared to control (between-group difference: -0.7 [95% CI -1.2, -0.2] mg/l; *p* = 0.003; adjusted for baseline). Results were confirmed in sensitivity analyses (Additional file 1).

### Changes in hs-CRP (baseline to 1 year)

**Fig. 2:**
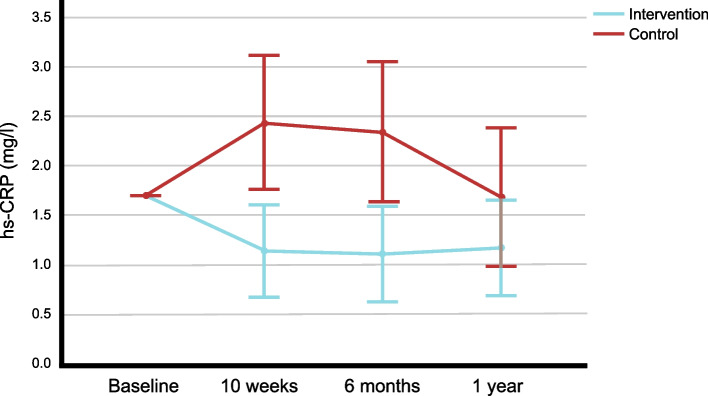
1-year trajectories of hs-CRP Values are means and 95% confidence intervals (adjusted for baseline); hs-CRP: high sensitivity C-reactive protein

From baseline to 1 year, hs-CRP significantly decreased in the intervention group (*p* = 0.002) and non-significantly decreased in the control group (*p* = 0.735; Table 2). The 1-year trajectory of hs-CRP was significantly lower in the intervention group compared to control (between-group difference: -0.8 [95% CI -1.2, -0.3] mg/l; *p* = 0.001; adjusted for baseline; Fig. 2). This result remained significant after Holm-Bonferroni correction. Adjusting for baseline hs-CRP, age, sex, education level, marital status, smoker status, alcohol intake, BMI, and HbA1c confirmed this result (*p* = 0.001; Table 2; sensitivity analysis). Adjusting for baseline hs-CRP, age, sex, education level, marital status, and changes (Δ[baseline, 1 year]) in smoker status, alcohol intake, BMI, and HbA1c also confirmed this result (*p* = 0.006; Table 2; sensitivity analysis). Furthermore, this result was confirmed by a sensitivity analysis using log-transformed (lg10) hs-CRP values (*p *= 0.006; adjusted for baseline), a sensitivity analysis including participants with an infection or common cold (self-reported at any measurement time point; non-log-transformed: *p* = 0.007; log-transformed: *p* = 0.007; adjusted for baseline; intervention: *n* = 92; control: *n* = 53), a sensitivity analysis including the 1½-year measurement time points (*p* = 0.004; adjusted for baseline; intervention: *n* = 59; control: *n* = 29; CCA), a sensitivity analysis using imputed data (LOCF; *p* = 0.005; adjusted for baseline; intervention: *n* = 84; control: *n* = 49), and a further sensitivity analysis using imputed data (LOCF) as well as including the 1½-year measurement time points (*p* = 0.013; adjusted for baseline; intervention: *n* = 79; control: *n* = 49). Another sensitivity analysis, exchanging the time points for the control group to achieve a comparable sequence of seasons (spring, summer, autumn, autumn), confirmed the results, with lower hs-CRP in the intervention group (between-group difference: -0.8 [95% CI -1.3, -0.3] mg/l; *p* = 0.003; adjusted for baseline hs-CRP, age, sex, education level, marital status, smoker status, alcohol intake, BMI, and HbA1c; intervention: *n* = 66; control: *n* = 32).

**Table 2:** hs-CRP and Hcy changes from baseline to 1 year in evaluable participants (CCA)

Parameters	hs-CRP, mg/l				Hcy, µmol/l				Apn, µg/ml			
**Group**	**IN** (*n* = 70)		**CON** (*n *= 34)		**IN** (*n* = 68)		**CON** (*n* = 52)		**IN** (*n* = 80)		**CON** (*n* = 61)	
	Mean	SEM or 95% CI	Mean	SEM or 95% CI	Mean	SEM or 95% CI	Mean	SEM or 95% CI	Mean	SEM or 95% CI	Mean	SEM or 95% CI
**Baseline**	1.3	0.2	2.6	0.7	12.4	0.4	11.8	0.4	10.5	0.6	7.7	0.6
**10 weeks**	0.9	0.1	3.0	0.8	12.3	0.4	12.2	0.4	8.0	0.4	7.9	0.7
**6 months**	1.0	0.1	2.6	0.6	-		-					
**1 year**	1.0	0.2	2.1	0.6	11.4	0.4	10.4	0.4	-		-	
**Δ(baseline, 1 year)**	-0.3	-0.7, 0.1	-0.5	-1.7, 0.8	-1.0	-1.8, -0.1	-1.4	-2.0, -0.8	-		-	
p WG *	**0.002** ^a^		0.735 ^a^		0.060 ^a^		** < 0.001** ^b^		** < 0.001** ^a^		0.595 ^a^	
**p BG †**	**0.001** ^c^				0.439 ^c^				**0.004** ^c^			
**p BG † (multivariable-adjusted)**	**0.001** ^d^				0.912 ^d^				**0.002** ^f^			
	**0.006** ^e^				0.825 ^e^				**0.003** g			

*CCA* Complete case analysis, *hs-CRP* High-sensitivity C-reactive protein, *Hcy* Homocysteine, *Apn* Adiponectin, *IN* Intervention, *CON* Control, *SEM* Standard error of the mean, *CI* Confidence interval, *p W*G *p*-values for within-group changes from baseline to 1 year, *p BG* *p*-values for between-group differences in 1-year trajectories, *BMI* Body mass index;

* p-value for within-group comparisons by:

^a^ Wilcoxon test (two-sided)

^b^ paired t-test (two-sided)

† p-value for between-group comparisons by:

^c^ repeated measures ANCOVA, adjusted for the baseline values of the respective parameters

^d^ repeated measures ANCOVA, adjusted for baseline values (hs-CRP and Hcy, respectively) as well as baseline age, sex, education level, marital status, smoker status, alcohol intake, BMI, and HbA1c

^e^ repeated measures ANCOVA, adjusted for baseline values (hs-CRP and Hcy, respectively), baseline age, sex, education level, marital status as well as changes (Δ[baseline, 1 year]) in smoker status, alcohol intake, BMI, and HbA1c

^f^ one-way ANCOVA, adjusted for the baseline Apn, age, sex, education level, marital status, alcohol intake, smoker status, BMI, TC, HDL-C, insulin, diastolic BP, and RHR

^g^ one-way ANCOVA, adjusted for the baseline Apn, age, sex, education level, marital status, and changes in alcohol intake, smoker status, BMI, TC, HDL-C, insulin, diastolic BP, and RHR

While the 1-year trajectory of hs-CRP was significantly lower in the intervention group compared to control, it was also observed that, within the intervention group, a significant hs-CRP decrease from baseline to 1 year was only indicated by the p-value but not the confidence interval (-0.7, 0.1 mg/l; Table 2) and that the confidence intervals of hs-CRP levels at the 1-year timepoint of both groups overlap (Fig. 2).

### Changes in Hcy (baseline to 10 weeks)

From baseline to 10 weeks, Hcy did not significantly change in either the intervention (-0.4 [95% CI -1.3, 0.5] µmol/l; *p* = 0.366; *n* = 80) or control group (0.2 [95% CI -0.6, 1.1] µmol/l; *p* = 0.736; *n* = 61). There was no significant difference in Hcy changes between the intervention and control group (between-group difference: -0.2 [95% CI -1.3, 0.8] µmol/l; *p* = 0.656; adjusted for baseline).

This result was confirmed when adjusting for baseline Hcy, age, sex, education level, and marital status as well as alcohol intake, smoker status, and HbA1c (*p* = 0.450) or changes in alcohol intake, smoker status, and HbA1c (*p* = 0.259; Supplementary table 3, Additional file 1; sensitivity analyses). Furthermore, a sensitivity analysis using log-transformed (lg10) Hcy values confirmed this result (*p* = 0.570; adjusted for baseline).

### Changes in Hcy (baseline to 1 year)

From baseline to 1 year, Hcy non-significantly decreased in the intervention group (*p* = 0.060) and significantly decreased in the control group (*p* < 0.001; Table 2). The 1-year trajectory of Hcy was non-significantly higher in the intervention group compared to control (between-group difference: 0.2 [95% CI -0.3, 0.7] µmol/l; *p* = 0.439; adjusted for baseline). This result was confirmed when adjusting for baseline Hcy, age, sex, education level, and marital status as well as smoker status, alcohol intake, BMI, and HbA1c (*p* = 0.912; Table 2) or changes (Δ[baseline, 1 year]) in smoker status, alcohol intake, BMI, and HbA1c (*p* = 0.825; Table 2; sensitivity analyses). Furthermore, this result was confirmed by a sensitivity analysis using log-transformed (lg10) Hcy values (*p* = 0.393; adjusted for baseline) and a sensitivity analysis using imputed data (LOCF; *p* = 0.328; adjusted for baseline; intervention: *n* = 92; control: *n* = 74).

### Changes in Apn (baseline to 10 weeks)

From baseline to 10 weeks, Apn significantly decreased in the intervention group (-2.5 [95% CI -3.5, -1.5] µg/ml; *p* < 0.001; *n* = 80), with no significant changes in the control group (0.2 [95% CI -0.5, 0.8] µg/ml; *p* = 0.595; *n* = 61). Apn changes were significantly lower in the intervention group compared to control (between-group difference: -1.6 (95% CI -2.7, -0.5) µg/ml; *p* = 0.004; adjusted for baseline). This result remained significant after Holm-Bonferroni correction. This result was also confirmed when adjusting for baseline Apn, age, sex, education level, and marital status as well as smoker status, alcohol intake, BMI, TC, HDL-C, insulin, diastolic BP, and RHR (*p* = 0.002) or changes in smoker status, alcohol intake, BMI, TC, HDL-C, insulin, diastolic BP, and RHR (*p* = 0.003; Supplementary table 3, Additional file 1; sensitivity analyses). Furthermore, a sensitivity analysis using log-transformed (lg10) Apn values confirmed this result (*p* = 0.018; adjusted for baseline).

### Dietary changes

**Fig. 3:**
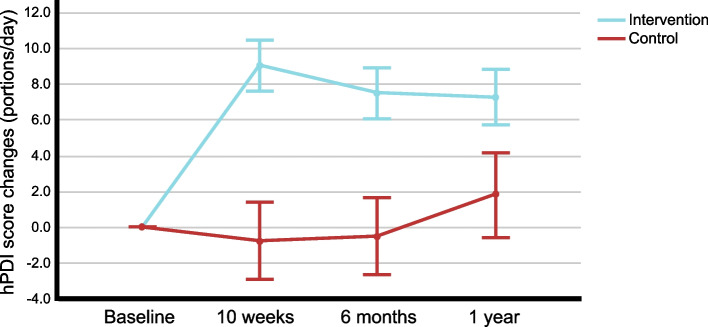
1-year trajectories of hPDI changes (from baseline; portions/day) Values are means and 95% confidence intervals (adjusted for baseline); hPDI: healthful plant-based diet index

PDI and hPDI changes from baseline to 10 weeks as well as the 6-month and 1-year trajectories were significantly higher (more favourable) in the intervention group, while uPDI changes were lower (more favourable) in the intervention group (all: *p *< 0.001; adjusted for baseline). 1-year trajectories of hPDI changes are shown in Fig. 3. Including the 1½-year time points confirmed these results (all: *p* ≤ 0.001; adjusted for baseline). The dietary intake data (including changes at the food group level) confirmed that participants of the intervention group were following the dietary recommendations given.

### Physical activity changes

Changes in physical activity from baseline to 10 weeks were significantly higher in the intervention group (intense: sessions/week, *p *= 0.006; moderate: minutes/week, *p *= 0.039; gentle: minutes/week, *p* = 0.042; adjusted for baseline values, age, and sex). However, no significant between-group difference was observed when changes in intense physical activity were assessed as minutes per week (*p* = 0.102). For the 6-month trajectories of physical activity, higher intense physical activity (when assessed as sessions/week; *p* = 0.012) and higher moderate physical activity (*p* = 0.049) were observed in the intervention group (adjusted for baseline values, age, and sex). For the 1-year trajectories of physical activity, no significant between-group differences were observed (adjusted for baseline values, age, and sex).

### Bivariate correlations of hs-CRP, Hcy, and Apn changes with changes in other markers

Bivariate correlations of changes in hs-CRP, Hcy, and Apn with changes in other CVD markers as well as changes in dietary scores are shown in Table 3. Only weak correlations were observed: 1-year hs-CRP changes positively correlated with body weight, BMI, waist circumference, and glucose and inversely correlated with Hcy. 1-year Hcy changes inversely correlated with REM-C (Table 3). 10-week Apn changes positively correlated with body weight, BMI, waist circumference, diastolic BP, TC, calculated LDL-C, non-HDL-C, REM-C, and insulin and inversely correlated with the plant-based diet scores PDI and hPDI (Table 3). At the food group level, most correlations between dietary intake and biomarkers were non-significant.

**Table 3:** Bivariate correlations of changes in hs-CRP, Hcy, and Apn with changes in other markers

**Parameter changes**	**Correlations with hs-CRP change** (Δ[baseline, 1 year])			**Correlations with Hcy change** (Δ[baseline, 1 year])			**Correlations with Apn change** (Δ[baseline, 10 weeks])		
	**r (CI)**	***p*-value**	**n**	**r (CI)**	***P* value**	**n**	**r (CI)**	***p*-value**	**n**
Body weight	0.231 (0.038; 0.408)	**0.018**	104	-0.132 (-0.305; 0.049)	0.152	120	0.254 (0.090; 0.405)	**0.002**	141
BMI	0.241 (0.048; 0.417)	**0.014**	104	-0.130 (-0.303; 0.051)	0.156	120	0.267 (0.103; 0.416)	**0.001**	141
WC	0.415 (0.234; 0.568)	** < 0.001**	104	-0.163 (-0.334; 0.019)	0.077	119	0.256 (0.092; 0.406)	**0.002**	141
Systolic BP	0.031 (-0.163; 0.222)	0.755	104	0.065 (-0.116; 0.242)	0.479	120	0.064 (-0.103; 0.228)	0.449	140
Diastolic BP	-0.075 (-0.264; 0.120)	0.448	104	0.006 (-0.173; 0.185)	0.945	120	0.183 (0.016; 0.340)	**0.031**	140
Pulse pressure	0.070 (-0.125; 0.259)	0.483	104	0.073 (-0.108; 0.249)	0.426	120	0.002 (-0.164; 0.168)	0.982	140
RHR	0.077 (-0.118; 0.266)	0.438	104	-0.100 (-0.275; 0.081)	0.276	120	0.019 (-0.147; 0.184)	0.821	140
TC	0.010 (-0.183; 0.202)	0.923	104	-0.009 (-0.188; 0.171)	0.923	120	0.251 (0.087; 0.402)	**0.003**	141
LDL-C (meas.)	-0.035 (-0.226; 0.159)	0.726	104	0.003 (-0.176; 0.182)	0.977	120	0.159 (-0.008; 0.317)	0.059	141
LDL-C (calc.)	0.029 (-0.165; 0.221)	0.767	103	-0.033 (-0.212; 0.148)	0.720	119	0.200 (0.034; 0.356)	**0.018**	140
non-HDL-C	0.045 (-0.149; 0.236)	0.653	104	-0.048 (-0.225; 0.132)	0.601	120	0.251 (0.087; 0.402)	**0.003**	141
REM-C	0.191 (-0.003; 0.372)	0.052	104	-0.186 (-0.355; -0.005)	**0.041**	120	0.203 (0.037; 0.358)	**0.016**	141
HDL-C	-0.138 (-0.323; 0.057)	0.161	104	0.075 (-0.106; 0.251)	0.416	120	0.133 (-0.034; 0.293)	0.115	141
TAG	0.137 (-0.058; 0.322)	0.167	104	-0.133 (-0.306; 0.048)	0.148	120	0.092 (-0.075; 0.254)	0.280	141
Glucose	0.216 (0.022; 0.394)	**0.028**	104	-0.083 (-0.259; 0.098)	0.370	120	-0.071 (-0.234; 0.096)	0.405	141
HbA1c	0.131 (-0.064; 0.316)	0.185	104	0.136 (-0.045; 0.308)	0.137	120	0.104 (-0.063; 0.265)	0.219	141
Insulin	0.066 (-0.128; 0.256)	0.503	104	-0.051 (-0.228; 0.130)	0.582	120	0.174 (0.008; 0.331)	**0.039**	141
hs-CRP	-	**-**	-	-	-	-	0.122 (-0.060; 0.296)	0.187	119
Hcy	-0.264 (-0.448; -0.059)	**0.011**	92	-	-	-	-0.052 (-0.216; 0.114)	0.542	141
PDI	-0.045 (-0.239; 0.153)	0.660	100	0.110 (-0.076; 0.289)	0.244	114	-0.178 (-0.342; -0.004)	**0.044**	129
hPDI	0.042 (-0.156; 0.237)	0.676	100	-0.064 (-0.245; 0.122)	0.497	114	-0.195 (-0.357; -0.021)	**0.027**	129
uPDI	-0.006 (-0.202; 0.191)	0.956	100	-0.116 (-0.294; 0.070)	0.218	114	0.134 (-0.041; 0.301)	0.130	129

Participants of both the intervention and control groups are combined. *hs-CRP* High-sensitivity C-reactive protein, *Hcy* Homocysteine, *Apn* Adiponectin, r: Spearman correlation coefficients, *CI* 95% Confidence interval, *BMI* Body mass index, *WC* Waist circumference, *BP* Blood pressure, *RHR* Resting heart rate, *TC* Total cholesterol, *LDL-C (meas.)* Measured LDL cholesterol, *LDL-C (calc.)* Calculated LDL-C, *non-HDL-C* Non-HDL cholesterol, *REM-C* Remnant cholesterol, *HDL-C* HDL cholesterol, *TAG* Triglycerides, *PDI* Plant-based diet index, *hPDI* Healthful PDI, *uPDI* Unhealthful PDI

## Discussion

In line with our study’s hypothesis, the 1-year trajectory of hs-CRP was significantly lower in the intervention group compared to control. Our results confirm that moving towards a healthier lifestyle (including a healthy plant-based dietary pattern) can decrease inflammation, as indicated by lower hs-CRP levels, even in subjects with low baseline levels [5]. In the intervention group, significant increases in physical activity were achieved at 10 weeks, but these were not maintained at 1 year. In contrast, dietary improvements were largely maintained at 1 year. A reason for this was likely that our intervention placed a strong emphasis on dietary recommendations. This also indicates that diet was likely a relevant factor in improving hs-CRP levels, which is in line with previously reported associations between healthy plant-based dietary patterns and lower hs-CRP [3, 7]. The observed 1-year improvement in hs-CRP of -0.8 mg/l (compared to control) indicates a clinically relevant effect [1] that is comparable to the effect of vegetarian diets documented in a recent meta-analysis (-0.6 mg/l) [7].

From baseline to 1 year, Hcy decreased in both the intervention (-1.0 µmol/l) and control group (-1.4 µmol/l; Table 2), with only a very small, non-significant difference between groups. While one may hypothesize that the control group may have had a slight advantageous effect due to a (hypothetically) higher intake of vitamin B12 (from animal-source foods) than the intervention group and a slightly increased intake of healthy plant foods (Fig. 3**)**, the observed results do not confirm this. Rather, the results appear to be in accordance with our study hypothesis and indicate that the recommendations given in the HLCP lifestyle programme did not adversely affect vitamin B12 status, as indicated by Hcy levels within 1 year.

However, we also observed an inverse correlation between Hcy changes (Δ[baseline, 1 year]) and changes in hs-CRP (Table 3). One could hypothesize that stronger adherence to plant-based dietary recommendations may more effectively lower hs-CRP but may at the same time adversely affect Hcy (due to potentially decreased vitamin B12 intake [16]). There is a lack of medium-term (≥ 1 year) controlled trials assessing the effect of lifestyle changes including a plant-based diet on Hcy levels in participants from the general population and using a no-intervention control group. One short-term controlled trial with these characteristics could be identified. This study, with healthy participants in Germany, demonstrated no effect of an unsupplemented vegan diet on Hcy levels after 4 weeks [38]. Controlled trials are needed to assess whether adopting a largely plant-based diet is associated with an increase in Hcy levels in the medium and long term. At 1 year, our intervention group had a mean Hcy plasma level of 11.4 µmol/l (Table 2). While some consider that Hcy values above 10 or 11 µmol/l may justify Hcy-lowering intervention [39], there is no consensus on adequate Hcy cut-off values, and a cut-off level of 14–15 µmol/l is also frequently used [14, 40]. Increased Hcy levels are associated with chronic disease risk [40]. Therefore, dietary recommendations should include strategies to ensure adequate intakes of vitamin B12, vitamin B6, and folate/folic acid.

While Apn significantly decreased in the intervention group from baseline to 10 weeks, Apn changes were not associated with changes in hs-CRP or Hcy (Table 3). However, Apn changes positively correlated with changes in body weight, BMI, waist circumference, cholesterol (TC, non-HDL-C, REM-C, calculated LDL-C), insulin, and diastolic BP and inversely correlated with changes in PDI and hPDI (Table 3). In observational studies, healthier, less inflammatory dietary patterns (including a traditional Mediterranean diet) tend to be associated with higher Apn levels, but these associations have not been consistently shown [41]. For example, vegetarian diets are not clearly associated with altered Apn levels [42].

In participants with metabolic syndrome, some lifestyle interventions have resulted in significant [32] (or non-significant [43]) Apn increases. However, apart from our study, no other controlled trials could be identified which have assessed the effect of a dietary or lifestyle intervention including a strong focus on a predominantly plant-based diet on Apn levels in mostly clinically healthy participants from the general population [31, 32]. Thus, our results cannot be compared to highly similar studies.

A recent review on dietary influences on Apn levels concluded that healthy dietary patterns (including a traditional Mediterranean or a Dietary Approaches to Stop Hypertension diet) as well as higher dietary intakes of fibre, monounsaturated and omega-3 fatty acids, polyphenols, alcohol, and dairy products are associated with higher Apn levels and that, in contrast, higher intakes of saturated and trans fatty acids, added sugars, and red meat as well as high glycaemic and high-carbohydrate low-fat diets are associated with lower Apn levels [44]. Our results appear to largely be in contrast with these findings. While our intervention advocated for moderation in alcohol and dairy intake, we did not observe significant correlations between Apn changes and changes in alcohol or dairy intake. In addition, other studies to date do not consistently confirm associations of Apn with alcohol or dairy intake: while a cross-sectional study with apparently healthy adults in Spain (aged ≥ 55 years) found a positive association of wine intake with Apn levels, there was no significant difference in Apn levels between alcohol abstainers and moderate drinkers in this study [45]. Similarly, a prospective cohort study with > 2800 participants in the United Kingdom (mean age: ~ 50 years) found that alcohol intake was not associated with Apn changes over time [46]. A recent meta-analysis of randomized controlled trials found that a high intake of dairy products was associated with higher Apn levels (~ 2.4 μg/ml higher compared with low or no dairy intake) [47]. However, other studies (not included in this meta-analysis) found that 400 ml/d of low-fat milk for 6 weeks had no significant effect on Apn (compared to control: habitual diet) [48] and that, in a 6-week crossover study, Apn significantly decreased in both the dairy intervention group (3.2 servings/d of 2% fat milk per 2000 kcal; ~ 11% Apn decrease) and the non-dairy control group (diet without dairy; ~ 13% Apn decrease), with no significant between-group difference [49]. In our study, Apn significantly decreased by ~ 24% in the intervention group (with no significant change in control; Supplementary table 3, Additional file 1). Furthermore, a recent study found that kefir or milk supplementation for 3 weeks did not significantly affect Apn (with no significant difference between kefir and milk) [50]. Thus, it appears uncertain whether changes in alcohol or dairy intake influenced our results to a relevant extent. While some studies have observed weight loss to be associated with an increase in Apn [42] and increased Apn levels have been observed in individuals with anorexia nervosa (in whom body fat mass is drastically decreased) [51], our study showed that a decrease in Apn was associated with a decrease in body weight, BMI, and waist circumference (Table 3). While the effects of exercise training on Apn are also uncertain [52], a slight majority of controlled trials with adults indicate that exercise is associated with significantly higher Apn levels [24]. In contrast, in our study, increased exercise levels after 10 weeks were associated with decreased Apn levels.

Results from Mendelian randomization studies suggest that blood Apn concentrations are unlikely to be causally associated with metabolic disease, including type 2 diabetes [53], coronary artery disease [54], and obesity-related cancer [55]. Based on Mendelian randomization, higher Apn levels may, however, adversely affect osteoarthritis risk [56] and bone mineral density (in the femoral neck and forearm) [57].

Taken together these results indicate that the decrease in Apn observed in our study may not constitute an unfavourable effect. Although it has been proposed that the Apn pathway is a highly relevant mediator of the beneficial effects of a healthy dietary pattern [58], currently the association of Apn with the beneficial effects of healthy lifestyle changes appears unclear [41]. Our results do not confirm the common interpretation that Apn increases observed in intervention studies constitute a beneficial effect. It should be noted that the correlations observed in the present study are weak (Table 3). As such, the observed correlations should be cautiously interpreted.

### Strengths and limitations

A strength of the present study is the use of a no-intervention control group (which allows for comparison with a group in which no effect is expected) and multiple measurement time points (which allowed us to confirm that hs-CRP was consistently more decreased in the intervention group at each follow-up time point). Two relevant limitations are the non-randomized design and the 6-month delay in starting the control group (although the follow-up durations were equivalent). While our findings indicate a more favourable 1-year trajectory of hs-CRP in the intervention group, this result may have been influenced by seasonal changes [59], although seasonal effects on hs-CRP are uncertain [60]. While a significant hs-CRP decrease from baseline to 1 year was observed in the intervention group and no significant change was observed in the control group (even though baseline levels in the control group were higher), seasonal influences remain a potential confounder. However, sensitivity analyses comparing 1½-year trajectories or exchanging the time points (of the control group) to achieve comparable seasons confirmed that the hs-CRP trajectory was significantly lower in the intervention group. This indicates that the results are not strongly confounded by seasonal effects. Although both groups were comparable at baseline and we adjusted for potential confounders, some bias due to non-randomization may have remained. Other limitations are the small study sample and the high proportion of participants who dropped out or were excluded from the analysis (although sensitivity analyses with imputed data confirmed the results).

## Conclusions

Over the course of 1 year, the lifestyle intervention lead to a significant improvement (decrease) in hs-CRP levels in a sample of individuals without strongly elevated baseline values, without adversely affecting Hcy. The widespread theory that an increase in Apn constitutes a beneficial health effect could not be confirmed in our study. Our results are in accordance with findings from recent Mendelian randomization studies which also indicate that this assumption should be reconsidered. Further studies should investigate how lifestyle interventions can be optimized to efficiently lower subclinical inflammation and thereby reduce disease risk. The parameters hs-CRP, Hcy, and Apn are secondary end points and our results should be considered exploratory.

## Supplementary Information

**Additional file 1:** **Supplementary table 1**. **Supplementary figure 1**. **Supplementary table 2**. **Supplementary table 3**.

## Data Availability

The dataset used during the current study is available from the corresponding author on reasonable request.

## Abbreviations

Apn: Adiponectin
BMI: Body mass index
BP: Blood pressure
CCA: Complete case analysis
CI: Confidence interval
CVD: Cardiovascular disease
Hcy: Homocysteine
HDL-C: HDL cholesterol
hPDI: Healthful PDI
hPDImod: hPDI modified
hs-CRP: High-sensitivity C-reactive protein
LDL-C: LDL cholesterol
non-HDL-C: Non-HDL cholesterol
PDI: Plant-based diet index
PP: Pulse pressure
PPARγ: Peroxisome proliferator-activated receptor-gamma
REM-C: Remnant cholesterol
RHR: Resting heart rate
SEM: Standard error of the mean
TAG: Triglycerides
TC: Total cholesterol
uPDI: Unhealthful PDI
