# Supplementary material for: Exploratory analysis of the effect of a controlled lifestyle intervention on inflammatory markers – the Healthy Lifestyle Community Programme (cohort 2)
Source: BMC Nutr. 2023 Feb 6;9:25. doi: 10.1186/s40795-023-00684-2 (PMC9900566; doi:10.1186/s40795-023-00684-2)
Supplement: Supplementary file 1 — Additional file 1: Supplementary table 1. Supplementary figure 1. Supplementary table 2. Supplementary table 3. [file 40795_2023_684_MOESM1_ESM.docx]

Additional file 1

# **Supplementary table 1**

| **Supplementary table 1:** Assessment methods and laboratory assays | | | |
| --- | --- | --- | --- |
| **Parameters** | **Serum/Plasma** | **Methods** | **Equipment** |
| hs-CRP | Serum | Spectrometry: immunonephelometry | Siemens BN 2 |
| Homocysteine | EDTA plasma | Competitive immunoassay | Siemens Immulite 1000 |
| Adiponectin | EDTA plasma | Enzyme-linked immunosorbent assay (Human Total Adiponectin/Acrp30 Quantikine ELISA; R&D Systems) | Grifols Diagnostic Triturus |
| All blood samples were taken in the morning and in the fasted state and were analysed at the University Hospital of Münster (Germany). The same standard operating procedures were followed for blood collection, processing, and storage during all study phases. Venous fasting (overnight) blood samples were taken by nurses. After waiting for ~15 min, samples were centrifuged, after which serum/EDTA plasma was separated from the centrifugate. Samples were stored at -80°C. All assays were performed in the same laboratory.  hs-CRP: high-sensitivity C-reactive protein | | | |

# **Supplementary figure 1**


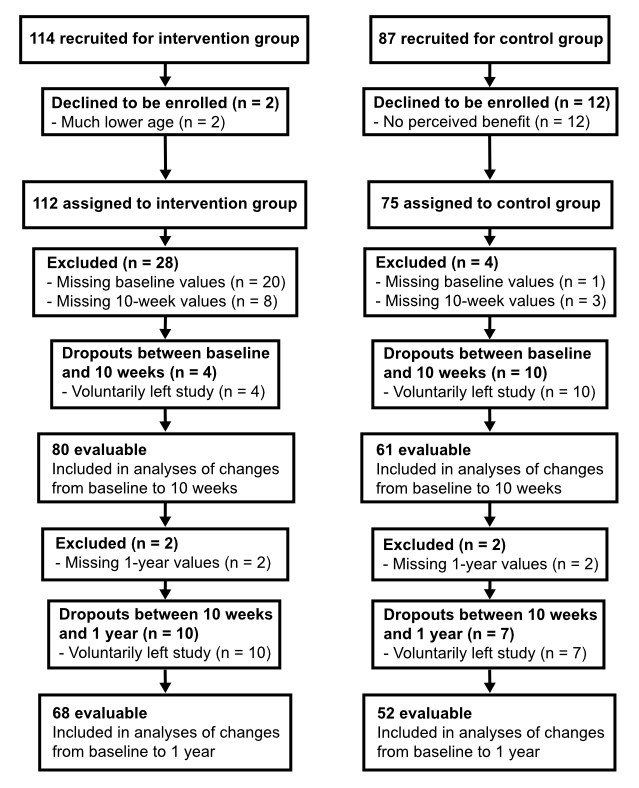


**Supplementary figure 1.** Flow chart of participants through the study

(homocysteine and adiponectin analysis)

# **Changes in hs-CRP (baseline to 10 weeks): sensitivity analyses**

Adjusting for potential confounders (baseline hs-CRP, age, sex, education level, marital status, alcohol intake, smoker status, BMI, REM-C, HDL-C, HbA1c, systolic BP, and resting heart rate (RHR)) confirmed this result (p = 0.002). Adjusting for baseline hs-CRP, age, sex, education level, marital status, and changes in alcohol intake, smoker status, BMI, REM-C, HDL-C, HbA1c, systolic BP, and RHR also confirmed this result (p = 0.004; **Supplementary table 2**). Furthermore, results were confirmed in a sensitivity analysis using log-transformed (lg10) hs-CRP values (p = 0.002; adjusted for baseline), a sensitivity analyses including participants with an infection or common cold (self-reported at either measurement time point; non-log-transformed: p = 0.036; log-transformed: p = 0.004; adjusted for baseline; intervention: n = 105; control: n = 62).

# **Supplementary table 2**

| **Supplementary table 2.** hs-CRP in evaluable participants at baseline and 10 weeks (CCA) | | | | |
| --- | --- | --- | --- | --- |
| **Parameter** | **hs-CRP, mg/l** | | | |
| **Group** | **IN** (n = 98) | | **CON** (n = 46) | |
|  | Mean | SEM or 95% CI | Mean | SEM or 95% CI |
| **Baseline** | 1.7 | 0.3 | 2.3 | 0.5 |
| **10 weeks** | 1.2 | 0.2 | 2.6 | 0.6 |
| **Δ(baseline, 10 weeks)** | -0.5 | -0.9, -0.1 | 0.3 | -0.6, 1.2 |
| **p WG** * | **<0.001** ^a^ | | 0.956 ^a^ | |
| **p BG** § | **0.006** ^b^ | | | |
| **p BG** § (multivariable-adjusted) | **0.002** ^c^ | | | |
|  | **0.004** ^d^ | | | |
| hs-CRP: high-sensitivity C-reactive protein; CCA: complete case analysis; IN: intervention; CON: control; SEM: standard error of the mean; CI: confidence interval; p WG: p-values for within-group changes from baseline to 10 weeks; p BG: p-values for between-group differences in changes from baseline to 10 weeks; BMI: body mass index; REM-C: remnant cholesterol; HDL-C: HDL cholesterol; BP: blood pressure; RHR: resting heart rate;  * p-value for within-group comparisons by:  ^a^ Wilcoxon test (two-sided)  § p-value for between-group comparisons by:  ^b^ one-way ANCOVA, adjusted for the baseline hs-CRP  ^c^ one-way ANCOVA, adjusted for the baseline hs-CRP, age, sex, education level, marital status, alcohol intake, smoker status, BMI, REM-C, HDL-C, HbA1c, systolic BP, and RHR  ^d^ one-way ANCOVA, adjusted for the baseline hs-CRP, age, sex, education level, marital status, and changes in alcohol intake, smoker status, BMI, REM-C, HDL-C, HbA1c, systolic BP, and RHR | | | | |

**Changes in hs-CRP (baseline to 6 months): sensitivity analyses**

Adjusting for baseline hs-CRP, age, sex, education level, marital status, smoker status, alcohol intake, BMI, and HbA1c confirmed this result (p = 0.002; sensitivity analysis). Adjusting for baseline hs-CRP, age, sex, education level, marital status, and changes (Δ[baseline, 6 months]) in smoker status, alcohol intake, BMI, and HbA1c also confirmed this result (p = 0.001; sensitivity analysis). Furthermore, this result was confirmed by a sensitivity analysis using log-transformed (lg10) hs-CRP values (p = 0.002), a sensitivity analysis including participants with an infection or common cold (self-reported at any measurement time point; non-log-transformed: p = 0.007; log-transformed: p = 0.002; adjusted for baseline; intervention: n = 99; control: n = 60), and a sensitivity analysis using imputed data (LOCF; p = 0.001; adjusted for baseline; intervention: n = 91; control: n = 55).

# **Supplementary table 3**

| **Supplementary table 3.** Hcy and Apn at baseline and 10 weeks in evaluable participants (CCA) | | | | | | | | |
| --- | --- | --- | --- | --- | --- | --- | --- | --- |
| **Parameters** | **Hcy, µmol/l** | | | | **Apn, µg/ml** | | | |
| **Group** | **IN** (n = 80) | | **CON** (n = 61) | | **IN** (n = 80) | | **CON** (n = 61) | |
|  | Mean | SEM or 95% CI | Mean | SEM or 95% CI | Mean | SEM or 95% CI | Mean | SEM or 95% CI |
| **Baseline** | 12.6 | 0.4 | 11.9 | 0.4 | 10.5 | 0.6 | 7.7 | 0.6 |
| **10 weeks** | 12.1 | 0.4 | 12.2 | 0.4 | 8.0 | 0.4 | 7.9 | 0.7 |
| **Δ(baseline, 10 weeks)** | -0.4 | -1.3, 0.5 | 0.2 | -0.6, 1.1 | -2.5 | -3.5, -1.5 | 0.2 | -0.5, 0.8 |
| **p WG** * | 0.366 ^a^ | | 0.736 ^a^ | | **<0.001** ^a^ | | 0.595 ^a^ | |
| **p BG** § | 0.656 ^b^ | | | | **0.004** ^b^ | | | |
| **p BG** § (multivariable-adjusted) | 0.592 ^c^ | | | | **0.001** ^c^ | | | |
|  | 0.450 ^d^ | | | | **0.002** ^f^ | | | |
|  | 0.259 ^e^ | | | | **0.003** ^g^ | | | |
| Hcy: homocysteine; Apn: adiponectin; CCA: complete case analysis; IN: intervention; CON: control; SEM: standard error of the mean; CI: confidence interval; p WG: p-values for within-group changes from baseline to 10 weeks; p BG: p-values for between-group differences in changes from baseline to 10 weeks; BMI: body mass index; TC: total cholesterol; HDL-C: HDL cholesterol; BP: blood pressure; RHR: resting heart rate;  * p-value for within-group comparisons by:  ^a^ Wilcoxon test (two-sided)  § p-value for between-group comparisons by:  ^b^ one-way ANCOVA, adjusted for the baseline values of the respective parameters  ^c^ one-way ANCOVA, adjusted for the baseline values of the respective parameters, age, and sex  ^d^ one-way ANCOVA, adjusted for the baseline Hcy, age, sex, education level, marital status, alcohol intake, smoker status, and HbA1c  ^e^ one-way ANCOVA, adjusted for the baseline Hcy, age, sex, education level, marital status, and changes in alcohol intake, smoker status, and HbA1c  ^f^ one-way ANCOVA, adjusted for the baseline Apn, age, sex, education level, marital status, alcohol intake, smoker status, BMI, TC, HDL-C, insulin, diastolic BP, and RHR  ^g^ one-way ANCOVA, adjusted for the baseline Apn, age, sex, education level, marital status, and changes in alcohol intake, smoker status, BMI, TC, HDL-C, insulin, diastolic BP, and RHR | | | | | | | | |
